# Friston's free energy principle: new life for psychoanalysis?

**DOI:** 10.1192/bjb.2021.6

**Published:** 2022-06

**Authors:** Jeremy Holmes

**Affiliations:** Department of Psychology, University of Exeter, UK

**Keywords:** Free energy, Friston, psychoanalysis, Freud, neuroscience

## Abstract

The free energy principle (FEP) is a new paradigm that has gain widespread interest in the neuroscience community. Although its principal architect, Karl Friston, is a psychiatrist, it has thus far had little impact within psychiatry. This article introduces readers to the FEP, points out its consilience with Freud's neuroscientific ideas and with psychodynamic practice, and suggests ways in which the FEP can help explain the mechanisms of action of the psychotherapies.

Today's psychiatrists are pragmatists, on the look-out for what ‘works’ and sceptical about the grand theories that held sway in the previous century. But ideology cannot be wholly avoided, nor theoretical controversy evaded. Current psychiatry's pantheon incudes evidence-based practice, DSM diagnosis and neuroscience. The search for evidence is theory driven. Diagnostic profusion raises questions about the medicalisation of human suffering. Despite extraordinary recent advances in neuroscience, their impact on everyday psychiatric practice has been modest.

The purpose of this article is twofold: first, to introduce readers to an overarching model of brain function associated with the mathematical psychiatrist Karl Friston, the free energy principle (FEP), which has been influential in neuroscience generally, but thus far has caused relatively little stir within psychiatry or clinical psychology. My hope is to redress that. Second, I make the case that FEP can revitalise the psychoanalytic psychotherapies, marginalised by the inexorable rise of cognitive–behavioural therapy (CBT) as the dominant psychological therapy paradigm.

It should be noted that FEP is deliberately described by Friston as a ‘principle’, akin to the principles of natural selection or gravity. The evidence for its validity is circumstantial rather than direct, and its detailed neuronal mechanisms and clinical implications remain to be fully explored.

## Friston's forebears

Friston's project builds on the work of a number of pioneering predecessors and their concepts. These include Erwin Schrödinger, Heinrich Helmholtz, the Claudes – Claude Bernard and Claude Shannon – and Thomas Bayes. We live in an entropic universe. Broken cups don't spontaneously reassemble. Coffee cools once poured. Stars burn out. The exception is life itself. Quantum physicist Schrödinger^1^ coined the term ‘negentropy’ to describe how living matter, Canute-like for its lifetime, reverses this cosmic tide towards disorder and homogeneity.

The key to negentropy is homeostasis. As Bernard famously put it, the condition of a free life is the stability of the interior milieu – whether one is a unicellular amoeba or, like Schrödinger, a Nobel-prize winning primate. Homeostasis, and the more general processes of allostasis,^2^ resist the forces of entropy, physiologically and behaviourally. Inherent in homeostasis are boundaries: cell membranes, the skin, the brain within its skull. Janus-like, homeostasis faces outwards towards the environment and inwards towards the *milieu interieur*. Temperature sensors in the skin tell us it's a hot day; the sympathetic nervous system activates sweat glands, the brain tells us to fling off jumpers, move into the shade, etc., all in the service of resisting being entropically fried. Note that homeostats vary in ‘precision’ – some are highly sensitive, whereas others tolerate a great range of variation.

Friston had the insight and mathematical sophistication to see that the negentropic homeostatic principle applies not just to the organism as a whole but to the brain itself.^3,4^ The brain's job is to counteract entropy and to maintain internal stability on behalf of the organism whose processes and behaviour it controls and directs; this applies, reflexively, to itself.

The FEP goes back to the ideas of 19th-century polymath Hermann von Helmholtz, updated by artificial intelligence (AI) neuroscientists Geoffrey Hinton and Peter Dayan.^5^ Naively, we tend to think of vision as a camera-like image passively projected onto the visual cortex, or the auditory system as microphone-like, responding indiscriminatingly to the prevailing phonic universe. In the Helmholtz model the *brain makes its own world*. Our sense organs, external and internal, are constantly bombarded by a vast range of stimuli from an ever-changing environment. To operate with maximum efficiency, the brain selects out the ‘meaning’ of its sensations, attending only to those that are relevant to its ‘affordances’^6^ – its specific ecological niche – and especially to input that is anomalous or novel.

Working in the 1950s at the Bell telephone company laboratory, Claude Shannon saw that this ‘meaning’ could be quantified – as ‘bits’ of information. Gregory Bateson, anthropologist and family therapy guru, called these ‘differences that make a difference’. White noise is chaotic, entropic and devoid of information. Language, whether spoken, sung or gestured, is structured, ordered, negentropic. The measure of informational energy is ‘surprise’, i.e. how unexpected a signal is. In the board game Scrabble, the letter ‘x’ conveys more information than ‘e’ because it is relatively unusual, applying to a smaller range of words, and so in calculating the score, is ‘worth’ more. The brain's aim is constantly to reduce informational entropy and maximise meaning.

A crucial building block for the FEP is the concept of the Bayesian brain. The Reverend Thomas Bayes, a late 18th-century clergyman and founder of probability theory, grasped, Doris Day-like, that the future's not ours to see. Yet, to survive and adapt we need to know, moment to moment, ‘what is going on’ – in ourselves, in the interpersonal world and in the physical world. On the basis of prior experience, the Bayesian brain^7^ continuously estimates the likelihood of future events. Probabilities are computed by comparing current states of affairs with past occurrences, estimating the extent of correspondence between them, factoring in the likelihood of errors in both memory and perception, and ending with a portion that represents that which cannot be predicted. This is ‘prediction error’, which must, in the service of negentropy, be minimised as far as is possible – prediction error minimisation or PEM.

The brain, ‘top-down’, uses Bayesian probabilities to clarify ‘bottom-up’ input, extero- and interocaptive:^8^ ‘My stomach is complaining, but it's not surprising – I overdid it on the pudding, so it's probably not cancer’; ‘I know that tune, I've heard it so many times – yes of course, it's the Beatles’ Yellow Submarine’; ‘Is that a stick or a snake? Come on, no adders in city centres, probably safe to pick it up’.

## Free energy

Now to the free energy principle itself. ‘Energy’ equates to information, albeit physically embodied in patterns of neuronal impulses, synaptic transmission (‘fire together, wire together’^9^) and the neurohormonal environment. Prior models of the world, top-down, ‘bind’ incoming bottom-up information. Energy *un*bound, or prediction error, reflects novelty in need of binding – and so forestall the dangers of entropic chaos.

Circumstantial evidence for the FEP is the fact that more neuronal fibres reach the eye downwards from the brain than travel upward towards the visual cortex. Whenever possible, the brain ‘tells’ the eye what it is likely to be seeing. The FEP postulates a hierarchical series of neuronal interactions, starting from the least to the most complex, from the periphery to the central nervous system, from specificity to abstraction, most of which operate below conscious awareness. At the level of the eye itself the retinal receptors are activated: ‘round, two dots and a straight line between’. Top-down, even in a 1-month baby, this will elicit an answering smile (‘face equals security’). Once language arrives, verbal concepts shape perceptions: ‘Oh of course, that's a face’. At the highest level is mentalising – thinking about thinking: ‘I wonder why bearded faces always make me feel slightly unsettled? Perhaps it's reminiscent of my scary grandfather’.

The FEP visualises a series of ‘conversations’ in which top-down ‘priors’ ‘bind’ bottom-up input into probabilistically recognisable meanings. Each level can be thought of as a meaning–action boundary. Ascending the hierarchy, the Bayesian process ensures that the most mathematically probable pattern prevails across these statistical boundaries or ‘Markov blankets’.^10^ Prediction error is minimised by ‘binding’ bottom-up energy (informational as well as physiological) by top-down generative models based on pre-existing patterns and concepts. Thus is order preserved, entropy eschewed. We know what we like and, mostly, see what we want and expect to see.

But there will always be a discrepancy between our pre-existing models of the world and incoming sensations, an excess of energy that cannot be bound and will have to be passed onto the next level up of the hierarchy. Lockdown excepted, we don't live huddled in ‘dark rooms’.^11^ The environment is constantly in flux; we need to explore as much as conserve – to find new sources of food, suitable mates, interest and excitement. Surprise, calibrated by the brain as the discrepancy between expectation and incoming sensation, is a proxy for free energy – and hence entropy. Surprise is both vital to survival but also potentially entropic, disruptive or even life-threatening. This represents the prediction error aforementioned. The brain minimises such surprise/error by whatever means possible.

At this point the role of affect becomes important. Free energy is aversive and can be thought of as representing mental pain. Conversely, ‘binding’ free energy is rewarding and therefore motivating. The role of affect, positive and negative, is to drive the free energy minimising processes. This is another ‘AI’ – active inference.

The idea of active inference captures a number of psychological processes central to psychological health. First, action or agency. Given that incoming stimuli are inherently subject to error and imprecision, the brain increases precision by movement – approaching an ambiguous stimulus source, turning the head to use foveal rather than peripheral vision, switching lights on in order to see better, etc. Second, top-down model revision. Now we know what that vague shape really ‘is’ – a cat, clothes strewn on the floor, etc.: ‘Let's listen more carefully. Oh, that's not the Beatles at all, it's the Beach Boys’. Third, and vitally in the case of social species such as our own, active inference is enhanced by recruiting help or ‘twogetherness’: ‘Did you hear something, or was I just imagining it?’; ‘You know about ’70s music – what was that group's name?’. Friston & Frith call this ‘duets for one’ and have worked out the mathematics of such collaborative Markov blankets.^12^ Fourth, if all else fails, by choosing or fashioning environments that conform to the brain's pre-existing models of the word: ‘I can't stand modern music. Let's go over to Classic FM’. This last aspect is captured by the psychoanalytic concept of ‘projective identification’, in which we shape our interpersonal world, often deleteriously, to conform with expectations: ‘You psychiatrists are all the same – never there when I need you’.

## Free energy and psychopathology

The FEP has clear implications for those who work in mental ill health, and especially who favour psychological methods of treatment. Consider depression, typically triggered by loss, trauma or multiple setbacks. Adversity is widespread – poverty, inequality, racism – but not all succumb. To understand resilience, we need an illness model that encompasses not just events, but individuals’ responses to them. Attachment research shows that those who are securely attached are able to repair the inevitable ruptures to which all are prone, often through the typical sequence of protest, rage, grief and mourning.^13^ As children, securely attached people have had caregivers they could depend on to acknowledge their pain, tolerate protest and help them to move on. Repeated episodes of everyday rupture–repair cycles help build this resilience.

The free energy released by the rupture is bound by the child's knowledge that help is at hand and that their epistemically trusted caregiver will provide a generative model to counteract the free energy associated with ruptures: ‘Don't worry love, I'm just going to the loo, I'll be back in a minute’. In the ‘still face’ paradigm, parents are asked to freeze their facial expression for 1 minute while talking or playing with their child.^14^ Securely attached children continue actively to try to re-engage with their caregivers in the confident expectation that they will be ‘back soon’. For insecurely attached children, by contrast, rather than rupture–repair, cycles of rupture–despair or rupture–disappear are the norm. Their caregivers have either themselves been overwhelmed by their child's unhappiness and so despairingly abandon attempts to alleviate it; or repress the impact of the child's mental pain and so ‘disappear’ emotionally. Both leave the child alone to find ways to bind the free energy the rupture evokes. When their caregiver's face freezes they look away, become miserable and regressed, and often resort to self-soothing rituals such as rocking or emotional dissociation.

Such insecurely attached children are primed in later life for depression in response to loss or trauma or, in extreme cases, to developing post-traumatic stress disorder. The ingredients of free energy minimisation needed to maintain psychological equilibrium are for them problematic. Active inference is compromised. They tend to be passive rather than active. They stick with limited and simplistic and inflexible ‘top-down’ models such as ‘It's no use trying to make things better, it never works’ or ‘Feelings are dangerous, best to keep them buried’. They find it hard to trust people and so can't ‘borrow’ an intimate other's brain with which to process feelings and build up alternative ways of viewing the world.

## Psychotherapeutic implications

The most commonly used therapy for depression, CBT, attempts to address these deficiencies. Therapists encourage patients actively to test their negative ‘hypotheses’ by looking more closely at their experiences and by exploring alternative top-down models to account for them (‘Maybe my boyfriend didn't answer his phone because he'd run out of battery, not because he doesn't love me’). But CBT has its limitations. ‘Treatment-resistant depression’ is common.^15^ People with personality disorders do badly with standard CBT, often refusing to engage or dropping out.^16^ The FEP provides explanations for this. From an FEP perspective, one way to minimise free energy is to gravitate towards or engender environments that confirm one's view of the world, however negative. Depression relegates sufferers to emotionally impoverished relationships, stereotyped and simplistic top-down models, and thus becomes a self-fulfilling hypothesis, resistant to psychotherapeutic interventions. In addition, these negative top-down priors are ‘inferentially inert’, i.e. inaccessible for modification.

A degree of chaos/uncertainty/free energy needs to be tolerated before new generative models can evolve. Homeostatic imprecision needs to be tolerated for a while. The holding and ‘negative capability’ of the therapist's ‘borrowed brain’ paves the way for a more complex, nuanced top-down reset. Given that people with personality disorders notoriously find it difficult to trust others, the brevity and defocus on the therapeutic relationship in standard CBT limits the scope for such fundamental change.

Moving from depression to an FEP perspective on trauma, the latter creates an overwhelming influx of free energy for which there are no available top-down models with which to bind it. Thoughts of cruelty, neglect and abuse remain in the realm of the unthinkable and are therefore ‘defended against’ by repression or dissociation.^17^ However, when jointly considered – under a shared Markov blanket – these bottom-up unprocessed experiences can be bound with the therapist's encouragement and expertise into manageable narratives. However painful, they become less overwhelming, a source of new ways of thinking and psychic reorganisation. As the patient begins to feel that the therapist is safe, reliable, compassionate and empathic, so everyday ruptures – session-endings, holiday breaks and misunderstandings – are repeatedly repaired via model revision (‘Maybe the weekend break does not inevitably mean I'm forgotten’), and the trust this engenders can be generalised into the patient's everyday life.

We can see here how contemporary psychoanalytic psychotherapy and revitalised Freudian ideas resonate with the FEP. Freud started off his working life as a neurologist. Like Friston, he conceptualised the brain's aim as reducing psychic energy, typically through action and ‘word representations’ – i.e. transmuting free energy into thinkable thoughts. He saw unbound energy (which he later transmuted into ‘libido’) as potentially disruptive and responsible for the symptoms of psychological illness. Psychoanalysis was designed first to evoke and then to quieten this trauma-related unbound energy. To achieve this, three key psychoanalytic procedures are free association, dream analysis and analysis of transference.

The ‘virtual’ nature of the psychoanalytic relationship brings both top-down and bottom-up components of the FEP process into focus, enabling them to be mentalised rather than enacted. Free association taps into the mind's normally unvoiced upward-welling stream of consciousness, counteracting the elusiveness of affect seen in the rupture–despair/disappear attachment pattern. This enables the range of top-down responses to be enhanced and aversive free energy minimised. At the top-down level, in a process comparable to the immune system's lexicon of antigen-activated antibodies, dreaming is the means by which the mind generates a repertoire of narratives with which to bind the free energy which life's vicissitudes engender. Transference analysis turns the spotlight on the limited varieties of top-down narratives that sufferers use in their dealings with intimate others to minimise free energy. The enigmatic ambiguity of therapists’ persona enables patients to experience, reconsider and extend the top-down assumptions with which they approach the world of intimate others.

Psychoanalysis has tended to self-isolation, sequestrated from cross-fertilisation by other disciplines. The Friston–Freud consilience opens up new possibilities. Psychoanalytic and attachment-derived mentalisation-based therapy (MBT) is now established as a highly effective therapy for borderline personality disorder, previously considered untreatable.^18^ MBT leads to big reductions in medication use, suicide attempts, hospital admission and unemployment among people with borderline personality disorder, as compared with treatment as usual.

MBT is both practically and conceptually consistent with the FEM. It encourages patients (a) to identify the bottom-up feelings that fuel their self-injurious actions, (b) to pause and think of different ways of handling these, i.e. to tolerate a quantum of free energy with the help of the therapists’ ‘borrowed brain’ and (c) through mutual mentalising (therapist and patient together forming a neurobiological ‘bubble’) to generate more complex and adaptive models of the self and significant others. The result is manageable surprise: confounding sufferers’ negative assumptions about the world, becoming less overwhelmed by unbound affect (fewer ‘melt-downs’) and facilitating greater resilience.

## Conclusions

If rehabilitation of the psychoanalytic method in the light of the FEP comes as a pleasant surprise, this is consistent with its principles. As in Mark Twain's trope, rumours of psychoanalysis's death have been greatly exaggerated. In place of despair or disappearance, the FEP suggests that repair is possible. FEP-grounded psychoanalytic approaches such as MBT are now known to help those with profound mental distress. They also suggest a scientifically sound account of the interpersonal and neuronal mechanisms by which psychological change comes about.

## About the author

**Jeremy Holmes** is a retired psychiatrist and psychoanalytic psychotherapist. He is a Visiting Professor at Exeter University, UK, and author of many articles and books in the field of attachment theory and psychoanalysis, including *The Brain Has a Mind of Its Own: Attachment, Neurobiology, and the New Science of Psychotherapy*, in which the ideas of this article are explored in greater detail.
